# Cecropin B Represses CYP3A29 Expression through Activation of the TLR2/4-NF-κB/PXR Signaling Pathway

**DOI:** 10.1038/srep27876

**Published:** 2016-06-14

**Authors:** Xiaoqiao Zhou, Xiaowen Li, Xiliang Wang, Xiue Jin, Deshi Shi, Jun Wang, Dingren Bi

**Affiliations:** 1 State Key Laboratory of Agricultural Microbiology, College of Veterinary Medicine, Huazhong Agricultural University, Wuhan 430070, P.R. China; 2 Hubei Provincial Institute of Veterinary Drug Control, Wuhan 430068, P.R. China

## Abstract

Cecropins are peptide antibiotics used as drugs and feed additives. Cecropin B can inhibit the expression of CYP3A29, but the underlying mechanisms remain unclear. The present study was designed to determine the mechanisms responsible for the effects of cecropin B on CYP3A29 expression, focusing on the Toll-like receptors (TLRs) and NF-κB pathways. Our results indicated that the CYP3A29 expression was inhibited by cecropin B, which was regulated by pregnane X receptor (PXR) in a time- and dose-dependent manner. Cecropin B-induced NF-κB activation played a pivotal role in the suppression of CYP3A29 through disrupting the association of the PXR/retinoid X receptor alpha (RXR-α) complex with DNA sequences. NF-κB p65 directly interacted with the DNA-binding domain of PXR, suppressed its expression, and inhibited its transactivation, leading to the downregulation of the PXR-regulated CYP3A29 expression. Furthermore, cecropin B activated pig liver cells by interacting with TLRs 2 and 4, which modulated NF-κB-mediated signaling pathways. In conclusion, cecropin B inhibited the expression of CYP3A29 in a TLR/NF-κB/PXR-dependent manner, which should be considered in future development of cecropins and other antimicrobial peptides.

In the past decades, infections of several pathogens have resulted in severe economic loss in the global pig industry^1^,^2^,^3^. The widespread use of antibiotics has resulted in increased antibiotic resistance, prompting the need for novel antimicrobial agents^4^,^5^. Several antimicrobial peptides (AMPs) and derivatives are currently in clinical development, mainly as topical agents^6^,^7^. They have been shown to have positive effects on performance, nutrient digestibility, intestinal microflora, morphology, and immune function in pigs and other animals^8^,^9^. Cationic AMPs, effectors of innate immunity contributing to first-line host defense, are emerging as innovative anti-infective agents^10^. Cecropin B, a natural cationic AMP produced by silkworms, has been shown to have the highest level of antimicrobial activity among the cecropin family^11^ and is considered as a valuable peptide antibiotic^12^. Previous studies have demonstrated that cecropins have anticancer activity^13^ and have been successfully commercialized as chemotherapeutic drugs^14^.

With the increasing use of cecropins for various purposes, there is an urgent need for a better understanding of their effects on the P450 metabolic pathways which are often responsible for drug metabolism and drug-drug interaction. Amon the P450 family, CYP3A4 is the predominant isoform in the hepatic-intestinal system and is responsible for metabolizing more than 50% of clinically used drugs in humans^15^,^16^,^17^. In preclinical setting, pig is becoming an important animal model in the evaluation of new drugs due to its physiological and anatomical similarity to humans^18^. The similarity of the primary structures of pig CYP3A29 and human CYP3A4 suggests that pig CYP3A29 is a good experimental model for the metabolic studies of drugs metabolized by human CYP3A enzymes^19^,^20^,^21^,^22^,^23^,^24^. Alterations in CYP3A29 activity are of particular interest, because they make up 30% of the total CYP450 proteins in pig; and a small change in its enzyme activity may lead to a remarkable change in drug efficacy and safety^25^. However, there have been no reports on the molecular mechanisms underlying the effects of biological polypeptides on drug metabolism enzymes. The investigation on the effects of biological polypeptides on the regulation of CYP3A29 expression and function is critical to the understanding of factors affecting drug safety and efficacy.

The present study was designed to investigate the regulatory pathways in cecropin B-induced CYP3A29 expression. First, we speculated that cecropin B activates target cells and regulates gene expression through TLR/ NF-κB pathways. AMPs target specific receptors, enzymes or proteins^7^. Earlier reports have demonstrated that mouse beta-defensin 2 activates immature dendritic cells through its interaction with TLR4^26^. Human beta-defensin 3 activates antigen-presenting cells via TLR1 and TLR2 in an NF-κB dependent manner^27^. The TLR signaling leads to the activation of NF-κB transcription factors, resulting in a direct induction or inhibition of the expression of downstream genes. Second, it has been demonstrated that the pregnane X receptor (PXR) regulates human CYP3A4 expression by associating with its obligate partner RXR-α, and that the heterodimer binds to PXR responsive elements that contain a half-site AG (G/T) TCA^28^,^29^,^30^. Recent pharmacological studies have demonstrated that the transcriptional activation of CYP3A29 is mediated by nuclear receptor PXR^31^. In light of the role of PXR in regulating the CYP3A29, we sought to examine whether PXR plays a role in cecropin B activity. Third, PXR regulates innate immunity, increasing the gene expression of key pattern recognition receptors such as Toll-like receptors (TLRs). Therefore, the activation of TLR /NF-κB that results in the modulation of the transcriptional activity of multiple steroid/nuclear receptors may cause PXR functional changes^32^,^33^,^34^,^35^,^36^.

In the present study, we hypothesized that cecropin B regulates the expression of CYP3A29 by interacting with TLRs, leading to NF-κB activation and the modulation of the expression of downstream genes PXR. We have demonstrated that cecropin B repressed CYP3A29 expression through activation of the TLR2/4-NF-κB/PXR signaling pathway in HepLi cells. The results of our study could guide the therapy of metabolic diseases by targeting signal transduction molecules, providing a basis for the better use of AMPs in veterinary and human clinical medicines.

## Results

### Cecropin B reduces CYP3A29 mRNA in a PXR-dependent manner and increases the nuclear export of RXR-α in primary hepatocytes and HepLi cells

It has been reported that infections induce AMPs, and suppress the biotransformation of drugs and decrease the hepatointestinal capacity of drug clearance^37^. Based on those findings, we tested whether cecropin B suppresses the expression of CYP3A29 and related nuclear receptors.

**Figure 1:**
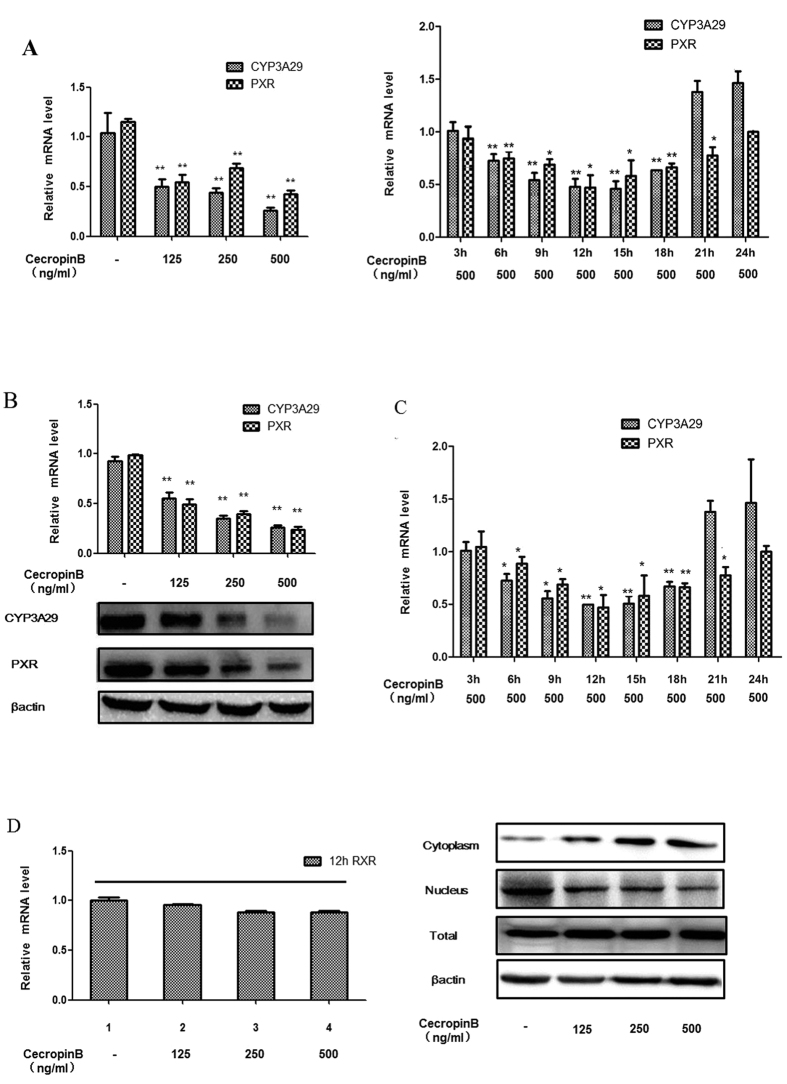
Effects of cecropin B on CYP3A29, PXR and RXR-α levels in primary pig hepatocytes and HepLi cells. (**A**) (left): Primary pig hepatocytes were treated with cecropin (**B**) (0, 125, 250 and 500 ng/ml) for 12 h. Relative mRNA levels of CYP3A29 and PXR were quantified by real-time RT-PCR. (**A**) (right): Primary pig hepatocytes were treated with 500 ng/ml cecropin B for different durations (3, 6, 9, 12, 15, 18, 21 and 24 hours). Relative mRNA levels of CYP3A29 and PXR were quantified by real-time RT-PCR. B: HepLi cells were treated with cecropin (**B**) (0, 125, 250 and 500 ng/ml) for 12 hours. Relative mRNA and protein levels of CYP3A29 and PXR were quantified by real-time RT-PCR and western blotting, respectively. C: HepLi cells were treated with 500 ng/ml cecropin (**B**) for different durations (3, 6, 9, 12, 15, 18, 21 and 24 hours). Relative mRNA levels of CYP3A29 and PXR were quantified by real-time RT-PCR. D: HepLi cells were treated with cecropin (**B**) (0, 125, 250 and 500 ng/ml) for 12 hours. Relative mRNA and protein (cytoplasm, nucleus and total) levels of RXR-α were quantified by real-time RT-PCR and western blotting, respectively; * and ** *P* < 0.01 and *P* < 0.05, respectively. Data are presented as mean and standard deviation (SD) of three independent experiments. The blot was cropped and the full-length blot is presented in Supplementary Fig. S2 (Fig. 1B,D).

We first examined the effect of cecropin B on cell viability in primary pig hepatocytes and HepLi cells (Supplemental Figure S1). The effects of cecropin B on the expression of CYP3A29, PXR and RXR-α were investigated in primary pig hepatocytes and HepLi cells by qRT-PCR and western blotting analyses. The cells were treated with cecropin B at various concentrations (0, 125, 250 and 500 ng/mL) for different durations (3, 6, 9, 12, 15, 18, 21, and 24 h) (primary pig hepatocytes Fig. 1A; HepLi cells, Fig. 1B,C). Results revealed that CYP3A29 and PXR levels significantly decreased, and the inhibitory effects of CYP3A29 and PXR were concentration- and time-dependent in short time. Notably, the CYP3A29 and PXR levels were increased after 12 h (Fig. 1A right panel and 1C). Although RXR-α mRNA levels did not significantly change after treatment with cecropin B, nucleus RXR-α levels were downregulated and cytoplasm RXR-α levels were upregulated in concentration-dependent manner (Fig. 1D); indicating that cecropin B increased the nuclear exportation of RXR-α into the cytoplasm.

**Figure 2:**
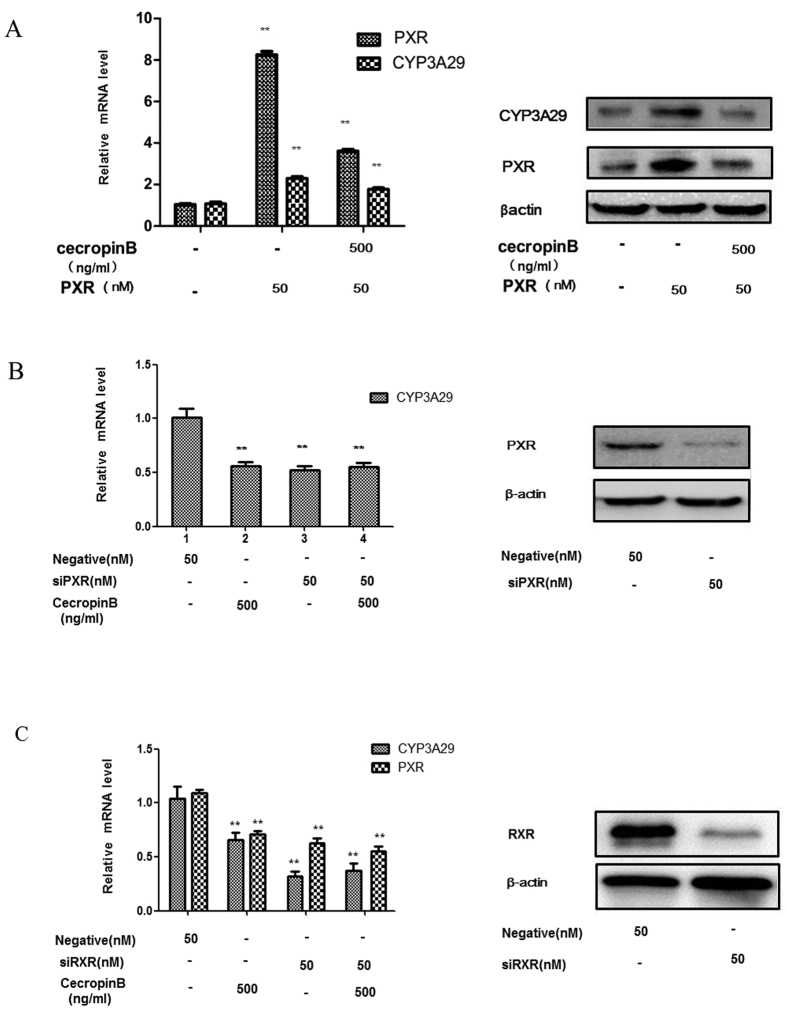
Regulation of CYP3A29 expression via PXR. (**A**) HepLi cells were transfected with 50 nM pcDNA-PXR expression plasmids or control vector for 24 h and treated with cecropin (**B**) (500 ng/ml) or PBS for 12 hours. CYP3A29/PXR mRNA and protein levels were detected by qRT-PCR and western blotting, respectively; * and ** *P* < 0.01 and *P* < 0.05, respectively. (**B**) HepLi cells were transfected with the PXR siRNA or the negative control for 24 hours and treated with cecropin (**B**) (500 ng/ml) or PBS for 12 hours. RNA interference efficiency was performed by western blotting(right). CYP3A29 mRNA levels were examined by qRT-PCR(feft). (**C**) HepLi cells were transfected with the RXR-α siRNA or the negative control for 24 h and then treated with cecropin B (500 ng/ml) or PBS for 12 h. RNA interference efficiency was determined by Western blotting (right). CYP3A29 and PXR mRNA levels were examined by qRT-PCR (left). The blot was cropped and the full-length blot is presented in Supplementary Fig. S2 (Fig. 2A–C).

To ascertain the effects of cecropin B via PXR and RXR-α, PXR transient transcription assays and PXR/RXR-α siRNA assays were performed. HepLi cells were transiently transfected with pcDNA-PXR expression plasmids and treated with cecropin B or PBS as control. Our results revealed that cecropin B significantly suppressed the upregulation of CYP3A29 and PXR expression by the overexpression of pcDNA-PXR expression plasmids (Fig. 2A). In the present study, siRNA successfully silenced the expression of endogenous PXR (Fig. 2B right). Compared with scrambled siRNA, the transfection with PXR siRNA effectively diminished the expression of CYP3A29; and the inhibitory effect of cecropin B on CYP3A29 was abolished as shown in Fig. 2B, column 4, compared with results shown in column 3 (Fig. 2B left). To further investigate the role of RXR-α in CYP3A29 and PXR expression, we performed RXR-α siRNA assays. As shown in Fig. 2C, RXR-α siRNA decreased more CYP3A29 level than cecropin B, and RXR-α siRNA restrained the inhibitory activity of cecropin B. Taken together, these results have demonstrated that cecropin B suppressed CYP3A29 via a PXR-dependent mechanism, and RXR-α was involved in this effect.

### Cecropin B represses the promoters of CYP3A29 and reduces PXR/RXR-α heterodimer binding to nuclear receptor response elements found in the regulatory regions of CYP3A29

**Figure 3:**
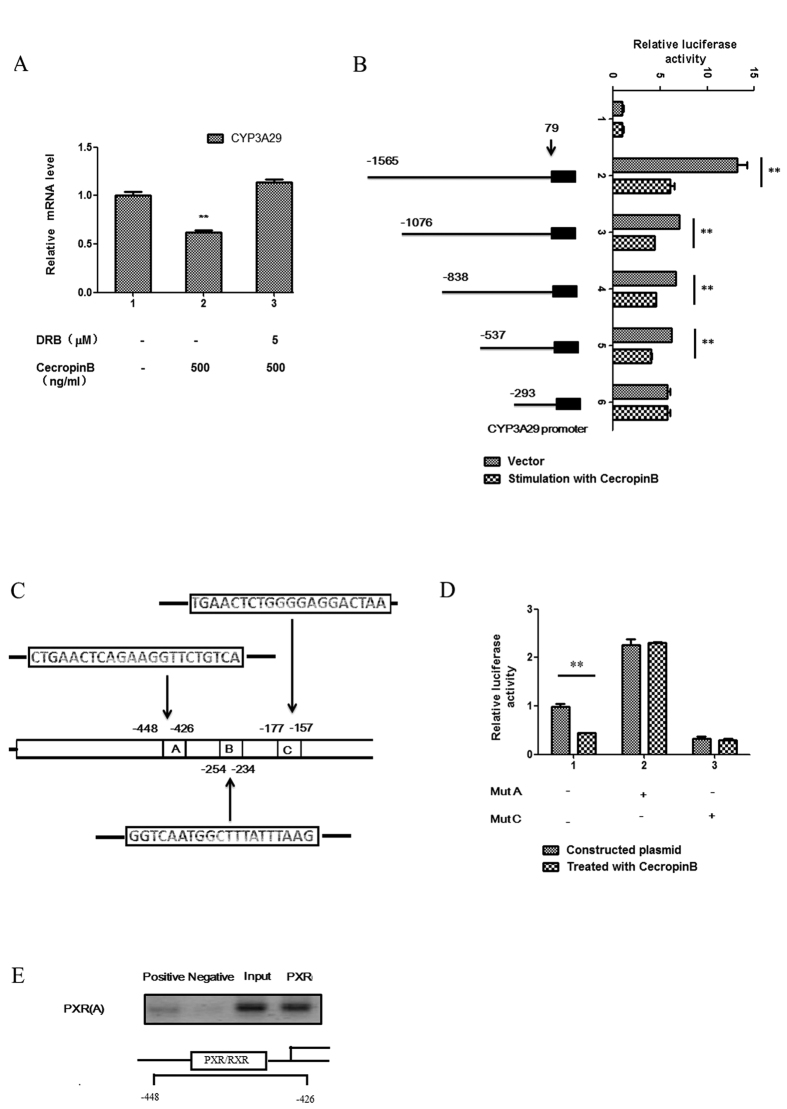
Effects of cecropin B on the transcriptional activity of CYP3A29 and PXR. (**A**) Cells were treated with 500 ng/ml of cecropin B for 12 hours in the absence or presence of 5 μM of DRB. Total RNA was prepared and CYP3A29 expression levels were analyzed by qRT-PCR. B: The luciferase reporter gene of CYP3A29 for cecropin B-regulated gene expression analysis. (**B**), left) HepLi cells were transiently cotransfected with CYP3A29 promoter reporter plasmids. (**B**), right) After 24 hours, transfected cells were treated with cecropin B. Luciferase activity was assayed 12 hours after treatments; ** and * *P* < 0.01 and *P* < 0.05, respectively. (**C**) Prediction of the PXR/RXR-α binding site in the CYP3A29 promoter region using JASPER software. D: HepLi cells were transiently cotransfected with site-specific mutant CYP3A29 promoter reporter plasmids. After 24 hours, transfected cells were treated with cecropin (**B**). Luciferase activity was assayed at 12 hours after the treatments. E: ChIP assay was performed using chromatin from Hep Li cells and anti-RNA polymerase II (positive), normal mouse IgG(negative), anti-PXR(PXR) were used as the immunoprecipitating antibody to immune select for the DNA for protein of interest that is specifically complexed with it. Purified DNA and total DNA (input) were then analyzed by PCR using primers specific for the C region.

To further investigate the effects of cecropin B on the transcriptional activity of CYP3A29 and PXR, we performed a transcriptional inhibition assay using an inhibitor of RNA synthesis DRB. Our results indicate that DRB completely abolished the cecropin B-mediated downregulation of CYP3A29 expression in HepLi cells, suggesting that cecropin B downregulated the expression of CYP3A29 by inhibiting its promoter activity (Fig. 3A).

Next, we constructed the promoter luciferase reporter gene of CYP3A29 and PXR for further analyses. The promoters of human CYP3A4 and PXR have already been intensively studied and human PXR is known to transcriptionally activate CYP3A4. However, the transcriptional mechanisms of pig CYP3A29 and PXR remains unclear.

In the present study, we identified several 5-variable CYP3A29 transcripts upstream of the transcriptional start sites of the major transcript. To characterize the transcriptional activity of the CYP3A29 gene, a series of different reporter plasmid constructs containing successive deletions of the CYP3A29 gene 5-flanking region were generated in the luciferase reporter plasmid pGL3-Basic system (Fig. 3B). We also mapped the minimal essential region for promoter activity to a 293-bp region upstream of the transcription initiation site. Moreover, we used the JASPAR (http://jaspar.genereg.net/) software to predict the binding sites of PXR/RXR-α in the CYP3A29 promoter region (−1565 to 79), and found three possible binding sites: A, B and C (Fig. 3C); in which, sites A and C had higher scores. To identify the functional PXR responsible for cecropin B-mediated downregulation, the site-directed mutagenesis assay of the PXR binding site was performed. Transactivation assays with the A and C mutated promoters potential supports that the effects of cecropin B were mediated by PXR binding site C (Fig. 3D).Interestingly, the mutation of binding site A led to the upregulation of CYP3A29 expression, and its mechanism of action needs further research. A ChIP assay was performed to investigate whether PXR was truly bound to PXR binding site C. The result showed that PXR gene sequence was amplified in purified DNA immunoprecipitated by anti-PXR (Fig. 3E). Our results revealed that the transcriptional regulations of pig CYP3A29 and PXR may help elucidate the regulatory mechanisms of pig drug metabolism.

### Cecropin B induces NF-κB activation

**Figure 4:**
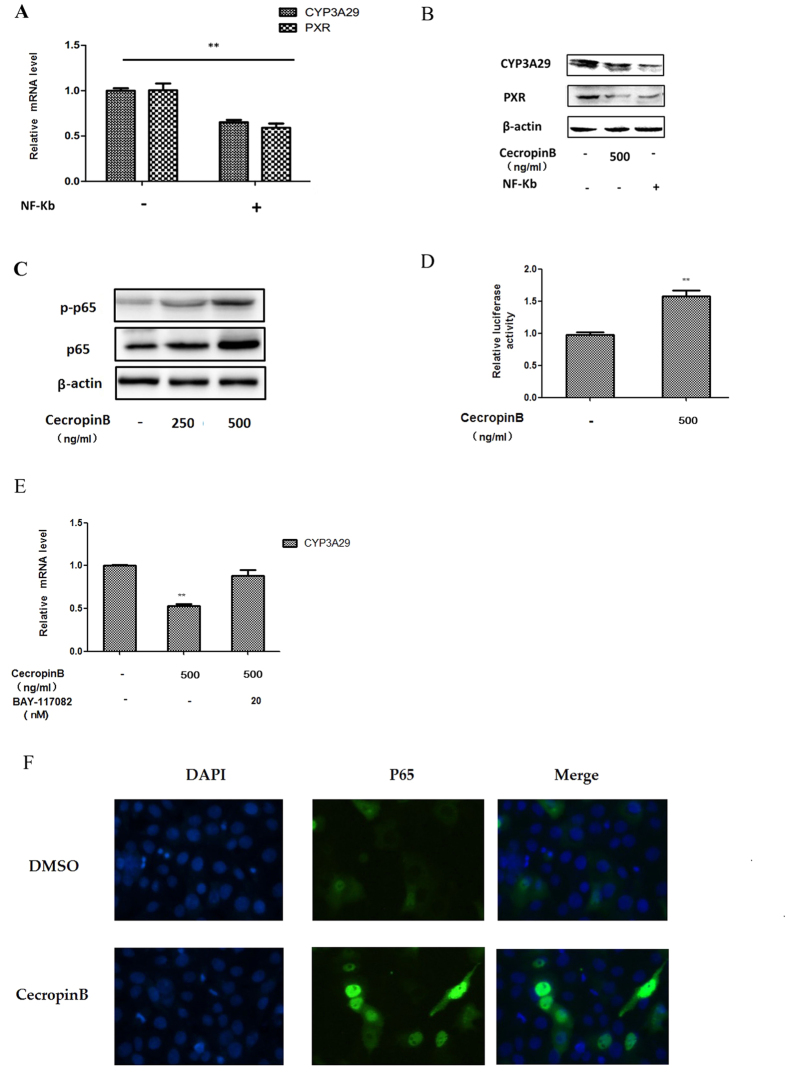
NF-κB activation is induced by cecropin B. (**A**) HepLi cells were transfected with the NF-κB p65 over expression vectoror control vector for 24 hours, and CYP3A29/PXR mRNA level were detected by qRT-PCR. (**B**) HepLi cells were treated with 500 ng/ml of cecropin B or transfected with the NF-κB p65 over expression vector, and CYP3A29/PXR protein levels were detected by western blot. (**C**) HepLi cells were treated with 250 and 500 ng/ml of cecropin B for 12 hours, and NF-κB p65 and NF-κB p-p65 protein levels were detected by western blot. (**D**) HepLi cells were transfected with the NF-κB reporter plasmid, and cells were treated with or without 500 ng/ml of cecropin B after 24 hours. Luciferase activity was assayed at 12 hours after the treatments. E: HepLi cells were cotreated with 500 ng/ml of cecropin B and 20 nM of BAY117082 or 500 ng/ml cecropin B alone for 12 hours. The control group was treated with PBS. F: HepLi cells were transfected with the NF-κB p65-GFP overexpression vector for 24 hours, treated with 500 ng/ml of cecropin B for one hour, and fluorescence was examined under an OLMLPUS IX51 inverted fluorescence microscope. ** and * P < 0.01 and P < 0.05, respectively. The blot was cropped and the full-length blot is presented in Supplementary Fig. S2 (Fig. 4B,C).

We transiently transfected NF-κB p65 overexpression vector (Fig. 4A) into cells to determine the role of NF-κB in mediating the suppression of CYP3A29 transcriptional inhibition. Our results revealed that NF-κB inhibited the expression of CYP3A29 and PXR, which were the same as cecropin B (Fig. 4B). One possible mechanism is that cecropin B can induce NF-κB activity. We speculated two possible regulation ways: inducing the expression of p65 and the nuclear translocation of p-p65. To test the hypothesis, we detected NF-κB (p65 and p-p65) protein levels after cecropin B treatment in HepLi cells. Our results indicated that the phosphorylation of p65 was upregulated, indicating that cecropin B may induce the nuclear translocation of p-p65, and that the total protein level of p65 was increased, indicating that cecropin B may induce the expression of p65 (Fig. 4C). Then, to confirm the above results, we transiently transfected the NF-κB p65-driven luciferase reporter gene into HepLi cells (Fig. 4D), the expression of p65 was up-regulated by cecropin B (Fig. 4D); and when specific inhibitor BAY11-7082 that could inhibit nuclear translocation of NF-kB was used, the suppression ability of cecropin B on CYP3A29 was significantly attenuated (Fig. 4E). These results indicated the critical role of NF-κB in the effects of cecropin B. Furthermore, the activation of NF-κB by cecropin B was confirmed by IHC for p65 nuclear translocation (Fig. 4F). These results supported the notion that NF-κB activation was directly responsible for cecropin B -regulated gene expression.

### Cecropin B induces NF-κB/PXR pathway activation

**Figure 5:**
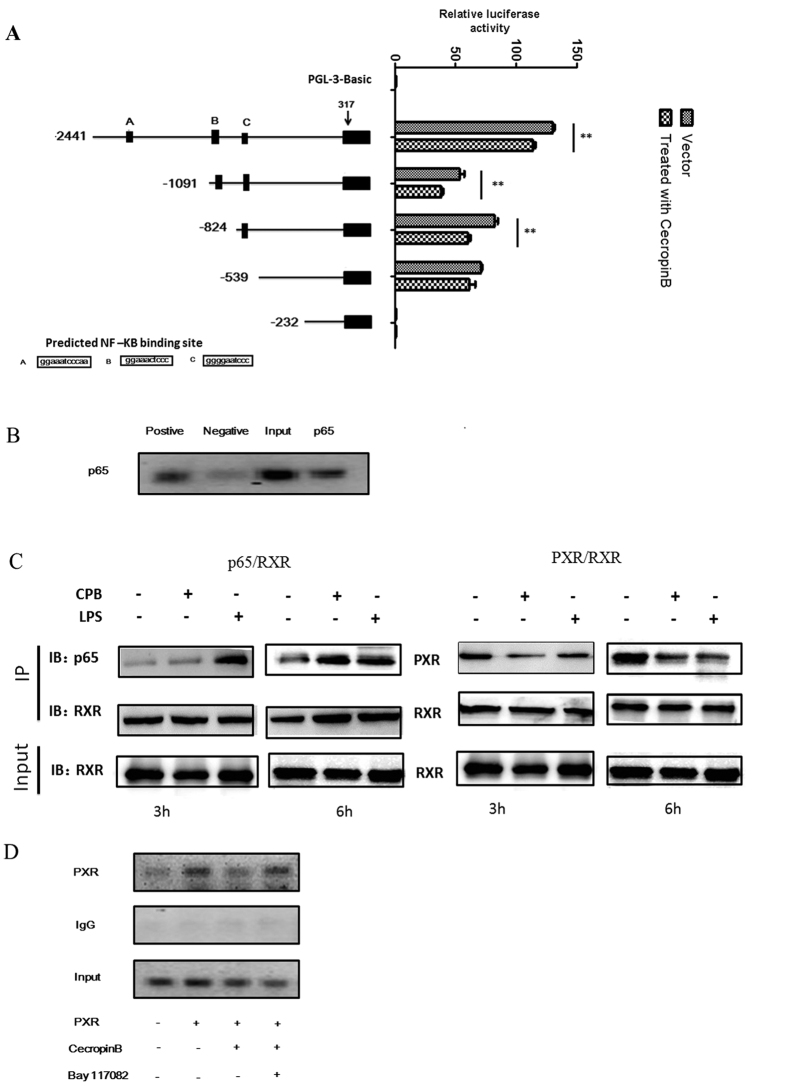
NF-κB/PXR pathway is activated by cecropin B. (**A**) The luciferase reporter gene of PXR for the analysis of cecropin B-regulated gene expression. HepLi cells were transiently transfected with PXR promoter reporter plasmids (left) and transfected cells were treated with cecropin B after 24 hours (right). Luciferase activity was assayed at 12 hours after the treatments; ** and *, statistically significant difference (*P* 0.01 and *P* 0.05, respectively). (**B**) ChIP analysis of HepLi cells fixed for 10 minutes; and DNA immunoprecipitated by p65 antibodies, immunoglobulin G, and RNA Polymerase II antibodies amplified by PCR. (**C**) HepLi cells were treated with cecropin B for three and six hours. CoIP assay was performed with anti-p65 and anti-PXR antibodies, followed by immunoblotting with anti-RXR-α antibody. LPS was used as positive control. D: HepLi cells were transfected with PXR over expression vector or control plasmid. After 24 hours HepLi cells were treated with cecropinB and Bay-117082 alone or together for 12 hours. The cells were formaldehyde cross-linked, and DNA immunoprecipitated by PXR antibodies, immunoglobulin G, and RNA Polymerase II antibodies amplified by PCR. The blot was cropped and the full-length blot is presented in Supplementary Fig. S2 (Fig. 5C).

It has been demonstrated that NF-κB regulates several nuclear/steroid receptors through physical and function interactions, resulting in the transrepression of gene expression regulated by these receptors^38^. In the present study, PXR was suppressed; and the expression of RXR-α was unchanged but exported into the cytoplasm (Fig. 1D). These results prompted us to hypothesize that cecropin B-induced NF-κB p65 activation might influence the physical association between the PXR/RXR-α heterodimer and NF-κB p65 subunit, or NF-κB inhibits the expression of PXR by binding to the promoter of PXR. To test this hypothesis, several fragments of the PXR promoter region were subcloned into a luciferase reporter vector and analyzed in transactivation assays. Three putative p65 sequences were identified in the PXR promoter (Fig. 5A). HepLi cells were transfected with pGL3-PXR 2441-Luc, pGL3-PXR 1091-Luc and pGL3-PXR 824-Luc; and exhibited a significant decrease in reporter activity by cecropin B treatment. This effect was not observed in pGL3-PXR 539-Luc and pGL3-PXR 232-Luc, and no basal activity was seen in pGL3-PXR 232-Luc (Fig. 5A). Notably, there were predicted p65 binding sites in pGL3-PXR 2441-Luc, pGL3-PXR 1091-Luc and pGL3-PXR 824-Luc reporter plasmids; but not pGL3-PXR 539-Luc and pGL3-PXR 232-Luc reporter plasmids. A ChIP assay was performed to identify the functional NF-κB responsible for PXR-mediated downregulation (Fig. 5B). Unexpectedly, we could only find one binding site: site C. These data clearly shows that NF-κB could bind to functional binding site C in the PXR promoter, indicating that the PXR promoter is a direct NF-κB target in pig liver cells.

Na *et al.* have reported that NF-κB directly interacts with RXR-α. The association of NF-κB with nuclear receptors may potentially have a functional impact on the transcriptional activity of the PXR/RXR-α complex. Therefore, we performed a co-immunoprecipitation assay to determine whether promoters targeted by PXR were disrupted by NF-κB. Results of the co-immunoprecipitation experiments are summarized in Fig. 5C. LPS was used as a positive control. NF-κB is an immediate early gene, which is activated in response to various stress stimuli. In the present study, a protocol of short-time treatment (three and six hours) was applied in the co-immunoprecipitation assay. In cells treated with cecropin B, the degree of the physical association between PXR and NF-κB p65 increased, while the interaction between PXR and RXR-α decreased, as shown in Fig. 5C. This result indicates that cecropin B inhibited CYP3A29 expression via promoting the physical interaction between RXR-α and the NF-κB p65 subunit, and reducing the interaction of the PXR/RXR-α complex with the CYP3A29 promoter. To further support these results, we examined the role of NF-κB on HepLi cells using ChIP assay (Fig. 5D). Cecropin B-treated cells revealed the attenuated binding level of PXR to the CYP3A29 promoter, which was enhanced after overexpression of the PXR expression plasmid. This marked decrease was eliminated by NF-κB inhibitor BAY117082. Thus, we could conclude that NF-κB has a critical role in the downregulation of CYP3A29 expression through the regulation of PXR transcriptional activity by disrupting the association between the PXR/RXR-α complex and DNA sequences. Simultaneously, cecropin B significantly activated NF-κB and reduced the expression of PXR target genes in cecropin B-stimulated HepLi cells.

### TLRs are required for the cecropin B-mediated inhibition of CYP3A29 gene expression

The components of microbial cell walls are potent activators of exogenous stimulus responses in animals^39^. Previous studies have indicated that human–defensin-3 activates antigen-presenting cells through TLRs 1/2 in an NF-κB-dependent manner^27^. In this study, we investigated whether TLRs are absolutely required for the inhibition of CYP3A29 genes in response to cecropin B in pig HepLi cells, and the mechanisms for controlling NF-κB signaling pathway activation.

**Figure 6:**
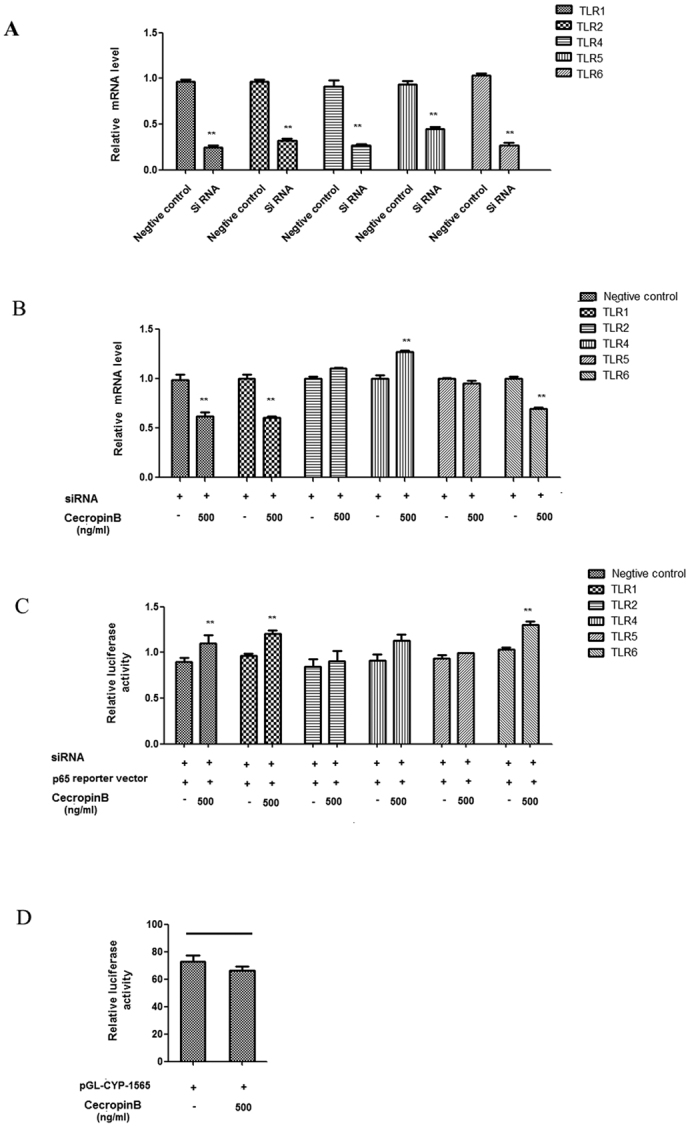
TLRs are required for the inhibition of CYP3A29 genes. (**A**) HepLi cells were transfected with the TLR 1/2/4/5/6 siRNAs and the negative control, respectively, for 24 h. RNA interference efficiency was demonstrated by qRT-PCR. (**B**) HepLi cells were transfected with siRNAs of TLR1/2/4/5/6 for 24 hours, and treated with 500 ng/mL of cecropin B for 12 hours. CYP3A29 mRNA levels were detected by qRT-PCR. (**C**) HepLi cells were co-transfected with siRNAs of TLR1/2/4/5/6 and NF-κB reporter plasmids for 24 hours. Then, cells were treated with 500 ng/mL of cecropin B. Luciferase activity was assayed at 12 hours after the treatments. (**D**) The 293T cells were transfected with CYP3A29 reporter plasmid for 24 hours, and treated with 500 ng/mL of cecropin B or control. Luciferase activity was assayed at 12 hours after the treatments. ** and * *P* < 0.01 and *P* < 0.05, respectively.

In the present study, siRNA assays of TLRs were performed by transfecting HepLi cells with TLRs 1, 2, 4, 5 and 6 siRNAs (the siRNA efficiencies were detected in Fig. 6A), followed by treatment with cecropin B or DMSO. As shown in Fig. 6B, silencing TLR2/4/5 attenuated the activity of cecropin B in the expression of CYP3A29. We also assessed the activation of p65 in HepLi cells co-transfected with TLR siRNA and NF-κB 65 reporter plasmid, followed by cecropin B treatment. TLR2/4/5 siRNAs reduced the activity of cecropin B in inducing NF-κB (Fig. 6C). Therefore, it may be inferred that TLR 2/4/5 are involved. To further confirm these results, we used 293T cells; since 293T cells do not express TLR2/4 (Fig. 6D), confirming that TLR2/4 were required.

## Discussion

In recent decades, there is an increasing interest in developing AMPs as natural antibiotics for preventing drug resistance development in infections and probiotic functions^40^. Previous studies have revealed that cecropin CD exhibit potent antiviral activity against PRRSV infection and replication *in vitro*^41^. Cecropin A is suggested to be a novel peptide antibiotic towards multidrug-resistant A. baumannii and P. aeruginosa^42^. Despite the potential of AMPs as a source of new drugs or feed additive, exploiting the field and its subsequent processes from preclinical studies to clinical applications is far from simple.

CYP3A29 is a predominant pig liver monooxygenase metabolizing more than half of the drugs in animal husbandry^31^. In the present study, we provided a molecular explanation linking cecropin B directly to the decreased activity of CYP3A29. Several mechanisms have been explored to explain the antimicrobial peptide-induced suppression of CYP3A29 expression.

In the present study, we demonstrated that the low physiological concentrations of cecropin B induced TLR-NF-κB responses in pig liver cells, resulting in the regulation and control of drug metabolism enzyme CYP3A29 responses associated with nuclear receptor PXR and RXR-α. The elucidation of the mechanism underlying the suppression of CYP3A29 by cecropin B was based on the following results. First, cecropin B treatments of pig hepatocytes resulted in the activation of NF-κB and coincided with the downregulation of CYP3A29/PXR. This result was consistent with previous results, in which the inhibition of TNF-α-induced NF-κB activation restores the expression of PXR and its target genes CYP3A4 and MDR1 in a PXR-dependent manner^43^. In the present study, we have shown that the expression pattern of CYP3A29 was highly correlated with that of PXR, indicating that the transcriptional regulation of PXR and CYP3A29 was similar to a previous report^31^. The inhibitory effects of cecropinB on the expressions of PXR and CYP3A29 were concentration- and time-dependent in short time, and with the treating time protracting, the mRNA levels of CYP3A29 and PXR stopped decreasing and gradually reversed (Fig. 1A right and Fig. 1C). Our results provided an explanation that as a drug metabolizing enzymes, CYP3A29 began to come back after the activity of enzyme was restrained for a period of time.

The transcriptional and posttranscriptional regulation of CYP3A29 expression is of great importance in therapeutic and feed additive applications, as well as in the development of novel therapeutics and animal feeds. It has been demonstrated that PXR regulates the expression of human CYP3A4 by associating with its obligate partner RXR-α, and the heterodimer binds to the nuclear receptor response elements found in the regulatory regions of downstream genes^44^. In our research, cecropin B had no effect on CYP3A29 mRNA level in the presence of PXR Si RNA (Fig. 2B) indicating that cecropin B regulates CYP3A29 via PXR. However, the regulation of CYP3A29 by RXR-α was unlikely via PXR alone. Herein we have identified RXR-α as a regulation factor for pig CYP3A29. The CYP3A29 reduction by RXR-α Si RNA was more strongly than that with cecropin B treatment (Fig. 2C). These results indicated a crosstalk between RXR-α and other nuclear receptors that might not be regulated by cecropin B. In the present study, there was an unexpected discovery that the nuclear export of the RXR-α protein was significantly increased with the increase in cecropin B concentration. We speculated that this effect on RXR-α was mediated by p65. Since PXR and RXR-α are major regulators of CYP3A^45^ and NF-κB is the central transcriptional regulator of immune and inflammatory responses^46^, we hypothesized that NF-κB inhibit the activity of PXR and RXR-α in pig liver cells. It is possible that NF-κB interferes with the association of PXR/RXR-α with DNA sequences and interacts with RXR-α, which may increase its nuclear export and separate the complex; causing the repression of CYP3A29. To gain more mechanistic insights, co-immunoprecipitation assay was performed. Our results have proven that the binding of PXR/RXR-α heterodimer to the PXR/RXR-α binding site was inhibited by p65, which was consistent with results from previous studies^37^. Furthermore, we performed ChIP assays to test whether cecropin B triggers the dissociation of PXR/RXR-α complexes to the binding site in the CYP3A29 promoter region. Our results demonstrate that cecropin B attenuated the formation of PXR/RXR-α complexes, affecting transcriptional regulation ability. As a pleiotropic transcription factor, NF-κB is activated in response to various stimuli^47^,^48^,^49^. In the present study, we have shown that cecropin B imposed the activation of NF-κB activity, leading to the repression of PXR. It is possible that the downregulation of PXR results in the downregulated expression of CYP3A29. In our luciferase reporter gene assay, the activation of NF-κB suppressed PXR driven luciferase reporter gene activity. Chromatin immunoprecipitation assay demonstrated that there was a NF-κB binding site in PXR promoter region that may cause the suppression expression of PXR. From these results, we can conclude that NF-κB regulates PXR through multiple mechanisms. However, due to extensive cross-talks, the compensatory roles of other nuclear receptors and nuclear transcription factors in mediating the cecropin B-induced suppression of gene expression remains to be investigated.

TLRs are thought to discriminate between self and non-self by recognizing highly conserved microbial patterns^50^,^51^, known as pathogen-associated molecular patterns (PAMPS). Infections and inflammatory responses have long been observed to suppress hepato-intestinal cytochromes P450, resulting in the reduced capacity of drug clearance in both humans and experimental animals, in which and the TLRs/NF-kB pathways play an important role^37^. In the present study, cecropin B decreased the capacity of CYP3A29 in pig liver cells in a TLR/NF-κB-dependent manner. It is noteworthy to mention that HepLi cells expressed low mRNAs levels of TLR1/5/6, which may be due to the clonal nature of the immortalized cell line. The separate interference of TLR2/4 induced the complete inhibition of cecropin B activity and enhanced activity of NF-κB, and there was no synergistic inhibition between them; which imply a co-dependence of TLR2/4. Thus, our studies provide evidence for a functional interaction between an exogenous peptide and TLRs. These data are consistent with previous studies that antimicrobial peptide regulates cell molecules maybe via a signal transduction pathway initiated by the engagement of the surface receptor complex based on TLRs^52^,^53^. It has been reported that papiliocin from swallowtail butter fly, Papilio Xuthus, shows high anti-inflammatory activity; and its innate defense response mechanisms engaged by papiliocin involve TLR pathways that culminate in the nuclear translocation of NF-κB^54^. Another finding has shown that interactions between viral particles and receptors on the cell membrane might be blocked by cecropin CD^41^. According to these reports, the inhibitory mechanism of cecropin B on CYP3A29 may indicate a competitive effect for TLRs between pathogen and cecropin B, even though there was no pathogen treatment in the present study. We speculate that cecropin B would provide a preventive effect for infection, which warrant further studies.

**Figure 7:**
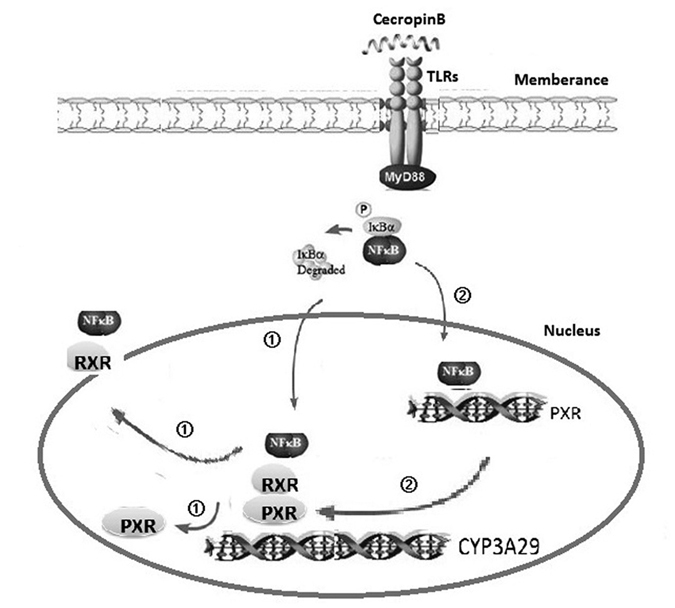
Schematic illustration of the possible mechanisms for the suppression of CYP3A29 gene expression by cecropin B. Upon activation of TLRs-NF-κB by cecropin B, NF-κB p65 translocates into the nucleus and disrupts the binding of PXR-RXR-α heterodimers to its regulatory sites by interacting with RXR-α, which is the obligate partner of PXR; thereby suppressing CYP3A29 expression. Simultaneously, NF-κB p65 binds to the promoter region of PXR, leading to the repression of PXR. The downregulation of the expression of PXR results in the downregulation of the expression of CYP3A29.

In summary, our data from the present study provide evidences supporting the notion that cecropin B can modulate both the transcriptional and posttranscriptional regulation of CYP3A29 through the TLR-NF-κB-PXR pathways. Possible mechanisms are outlined in Fig. 7. Our results would improve the understanding of the biological role of nuclear receptors and nuclear transcription factors, and help design new drugs to overcome the limitations of current therapies. Metabolism-related enzyme, transporter proteins and signal pathways can be regulated by the activated nuclear receptors, which may play important roles in gluconeogenesis, lipid metabolism and bile acid homeostasis^55^. In conclusion, cecropin B regulates nuclear receptors and nuclear transcription factors, and can be a potential drug for RXR-α and PXR-mediated metabolic diseases and cancers.

## Materials and Methods

### Cell Lines, Test drugs, Chemicals and Reagents

Pig hepatocyte cell line HepLi^31^ was a kind gift from Dr. Xiaopin Pan (State Key Laboratory for the Diagnosis and Treatment of Infectious Diseases, Collaborative Innovation Center for the Diagnosis and Treatment of Infectious Diseases, First Affiliated Hospital, School of Medicine, Zhejiang University, Hangzhou, China). Cecropin B and (E)-3-[(4-methylphenylsulfonyl]-2-propenenitrile, an NF-κB inhibitor, were purchased from Sigma-Aldrich (St. Louis, MO, USA). Monoclonal mouse antibody against p65 was obtained from Cell Signaling Technology (Boston, MA, USA), and polyclonal antibodies against PXR, RXR-α, and β-actin were purchased from Bioss Inc. (Beijing, China), and polyclonal antibody against CYP3A29 was obtained from Santa Cruz Biotechnology (Santa Cruz, CA, USA). Other reagents were purchased from Sigma-Aldrich unless otherwise described. The p65 promoter reporter construct and p65 expression vector encoding GFP were kind gifts of Dr. Rui Lo (State Key Laboratory of Agricultural Microbiology, College of Veterinary Medicine, Huazhong Agricultural University, Wuhan, China). Fetal bovine serum (FBS) and Dulbecco’s modified Eagle’s medium (DMEM) were purchased from Gibco Life Technologies (Grand Island, NY, USA). The dual-luciferase reporter assay system was obtained from Promega (Madison, WI, USA). Lipofectamine 2000 Transfection Reagent was purchased from Invitrogen (Life Technologies, Carlsbad, CA, USA).

### Cell Culture

HepLi cells were cultured in DMEM supplemented with 10% FBS and 1% Penicillin-Streptomycin antibiotics at 37 °C in a humidified incubator with 5.0% CO_2_. Hepatocytes were isolated from Chinese experimental miniature pigs using a modified four-step collagenase perfusion method. Our experimental protocols were reviewed and approved by the Animal Care Ethics Committee of our institution (approval number 42000400002483) and followed the Guidelines for Humane Care of Laboratory Animals. Our study was carried out in accordance with the approved guidelines of Animal Care Ethics Committee of HuaZhong Agricultural University. All efforts were made to minimize animal suffering during the study. Approximately 5 × 10^6^ primary hepatocytes /well were plated onto a 6-well plate with 3 ml of DMEM containing 20% FBS, insulin (10 mg/L), penicillin (100 U/mL) and streptomycin (100 μg/mL) in each well. HepLi cells were seeded at a density of 2 × 10^6^ cells/well in 6-well plates. Cecropin B treated cells were cultured in 2% serum-reduced medium supplemented with 10% FBS and 1% Penicillin-Streptomycin.

### Plasmid Constructs

The reporter plasmid pGL3–3A29-Luc was constructed in the following steps. First, the promoter module (−1565/79, −1076/79, −838/79, −537/79, −293/79) -containing DNA fragment was generated by PCR amplification using pig genomic DNA as the template with the primers shown in Supplemental Table S1. Then, PCR products were digested with restrict enzymes KpnI and XhoI, and the resultant fragments were cloned into the pGL3-basic vector (Promega) to yield pGL3–3A1565-Luc, pGL3–3A1076-Luc, pGL3–3A838-Luc, pGL3–3A537-Luc and pGL3–3A293-Luc.

The reporter plasmid pGL3–PXR-Luc was constructed via the following steps. The promoter module (−2441/317, −1091/317, −824/317, −539/317, −232/317) -containing DNA fragment was generated by PCR amplification using pig genomic DNA as the template with the primers shown in Supplemental Table S2. PCR products were restricted with XhoI and Hind III, and resultant fragments were cloned into the pGL3-basic vector (Promega) to yield pGL3–PXR2441-Luc, pGL3–PXR 1091-Luc, pGL3–PXR 824-Luc, pGL3–PXR 539-Luc and pGL3–PXR 232-Luc.

Bioinformatics analyses were performed to predict transcription factor binding sites using the web site, http://jaspar.genereg.net/. Polymorphic variants were constructed using the ClonExpress^®^ Entry One Step Cloning Kit (Vazyme, Nanjing, China). The construct and all of the mutants (Fig. 3C) were sequencing-confirmed. The pRL-TK Renilla reniformis luciferase plasmids (Promega) were used to normalize firefly luciferase activities.

### Transient Transfection

HepLi cells were seeded in 24-well plates and cultured until 80% confluence, and transfection was conducted by lipofection with Lipofectamine™ 2000 Transfection Reagent (Invitrogen, CA, USA). Transfection mixtures contained 500 ng of vector plasmid with promoter fragment and 25 ng of pRL-TK plasmid. Cells were transfected for six hours, and the medium was replaced with fresh medium containing cecropin B or DMSO. Transfected cells were incubated for another 12 hours and reporter enzyme activity was determined with a dual-luciferase reporter assay system (Promega), according to manufacturer’s instructions. Results were calculated from three or more independent transfection experiments.

For assays with PXR overexpression vectors, the cells were seeded in 6-well plates and transfected with mixtures containing 1.5 μg of PXR overexpression vector. Transfected cells were incubated for another 12 hours and harvested to determine mRNA and protein levels.

### Real-Time Quantitative PCR

Total RNA was isolated from cells with the TRIzol reagent (Invitrogen) and reverse transcribed (RT) with the Superscript reverse transcriptase (Takara, Otsu, Japan). The qRT-PCRs were performed using iQ^™^ SYBR Green PCR Supermix (Takara) in the Bio-Rad CXF real-time PCR detection system (Bio-Rad, California, USA). Primer sequences for pig CYP3A29, PXR and RXR-α are shown in Supplemental Table S3.

### Western Blot Analysis

Protein expression levels were determined using western blotting, as previously described^31^. In brief, total cellular proteins were extracted using RIPA Lysis Buffer (Pierce Biotechnology, Meridian Rd, Rockford, USA). BCA reagents (Pierce Biotechnology, Meridian Rd, Rockford, USA) were used to measure protein concentrations. Equal amounts of proteins (500 μg) were separated by 10% SDS-PAGE and transferred onto polyvinylidene fluoride membranes. The blots were immunoreacted with appropriate primary antibodies and horseradish peroxidase (HRP)-conjugated secondary antibodies, and the proteins of interest were visualized by the ECL chemiluminescence system (Bio-Rad). All the gels were run under the same experimental conditions.

### Chromatin Immunoprecipitation (ChIP) Assay

ChIP assays were performed using a previously reported method^37^. In brief, cells were transfected with the PXR expression vector or pcDNA3.1 empty vector for 24 hours; and the medium was replaced with fresh medium containing DMSO, cecropin B, or NF-κB inhibitor for 12 hours. Cells were cross-linked with 1% formaldehyde and quenched before harvest and sonication. The sheared chromatin was immunoprecipitated with anti-PXR or control IgG and protein G Sepharose beads. After washing with PBS, the beads were eluted with 50 μL of elution buffer (1% SDS, 100 mM of NaHCO_3_). Eluted immunoprecipitates were digested with proteinase K, and DNA was extracted and used for PCR with primers specific for pig CYP3A29 promoter regions. DNA samples were analyzed using quantitative PCR, and DNA binding level was expressed as fold enrichment above the control IgG. PCR primers were as follows: 5′-TCCTACAGAATATGAACTCTGG-3′ (forward) and 5′-TGCAGCCAATGGAAGAGC-3′ (reverse) for amplifying the CYP3A29 promoter region; 5′-ATTCCTCAGAGGGAGACAAA-3′ and 5′-CCGACCTCCATTCTACTC-3′ for amplifying the PXR promoter region.

### RNA Interference

SiRNA sequences targeting PXR, RXR-α and TLRs used in the present study are shown in Supplemental Table S4. SiRNA with scrambled sequence was used as negative control (NC siRNA). The double-stranded RNAs (50 nM) were transfected into cells with Lipofectamine 2000 (Invitrogen, California, USA). SiRNA experiments were performed based on a method previously reported^31^.

### Immunohistochemistry (IHC)

HepLi cells were grown in 24-well plates and transfected with the p65-GFP vector for 24 hours; then, cells were treated with cecropin B for one hour. The cells were washed thrice with cold PBS and fixed with fresh 4% formaldehyde in PBS at room temperature for 10 min. After washing with PBS thrice, the cells were permeablized with 0.2% Triton X-100 for 10 min at room temperature and stained with 4′-6-diamidino-2-phenylindole-dihydrochloride (DAPI, Invitrogen) to detect nuclei.

### Co-immunoprecipitation

The co-immunoprecipitation assay was conducted to examine the physical association between PXR and RXR-α/PXR and NF-κB p65 subunits using SureBeads™ Starter Kit Protein G (Bio-Rad), and detected with western blotting following manufacturer’s instructions.

### Statistical analysis

Data are presented as mean ± standard deviation (SD) of at least three independent experiments. Statistical analysis was performed using SAS software version 9.1 (SAS Institute, Cary, NC, USA). Statistical analysis was carried out using one-way analysis of variance (ANOVA), followed by Duncan’s multiple comparison tests. *P* < 0.05 was considered statistically significant.

## Additional Information

**How to cite this article**: Zhou, X. *et al.* Cecropin B Represses CYP3A29 Expression through Activation of the TLR2/4-NF-κB/PXR Signaling Pathway. *Sci. Rep.* **6**, 27876; doi: 10.1038/srep27876 (2016).

## Supplementary Material

Supplementary Information
