# Supplementary material for: Cecropin B Represses CYP3A29 Expression through Activation of the TLR2/4-NF-κB/PXR Signaling Pathway
Source: Sci Rep. 2016 Jun 14;6:27876. doi: 10.1038/srep27876 (PMC4906279; doi:10.1038/srep27876)

Supplementary Information

Cecropin B Represses CYP3A29 Expression through Activation of the TLR2/4-NF-κB/PXR Signaling Pathway

Xiaoqiao Zhou[a], Xiaowen Li[a], Xiliang Wang[a*], Xiue Jin[b], Deshi Shi[a], Jun Wang[b], Dingren Bi [a*]

[a] State Key Laboratory of Agricultural Microbiology, College of Veterinary Medicine, Huazhong Agricultural University, Wuhan 430070, P.R. China

[b] Hubei Provincial Institute of Veterinary Drug Control, Wuhan 430068, P.R. China

Corresponding author:

Dingren Bi, professor

State Key Laboratory of Agricultural Microbiology, College of Veterinary Medicine, Huazhong Agricultural University. Wuhan 430070, P.R. China

Tel: +86-27-87280208 Fax: +86-27-87280408

Email: bidingren@mail.hzau.edu.cn

Xiliang Wang, associate professor

State Key Laboratory of Agricultural Microbiology, College of Veterinary Medicine, Huazhong Agricultural University. Wuhan 430070, P.R. China

Tel.: (+86) 27-87280566; fax: (+86) 27-87280208

Email: wxl070@mail.hzau.edu.cn

Table S1-S4

Table S1. Sequences for primers used in PCR

Gene	Primers
-1565/79	Forward: 5’-GGTACCATGGTACCTCAGGCCAGAGGGAGAG-3’
	Reverse: 5’-CTCGAGGCCTCGAGACCGCTACTCACAGATA-3’
-1076/79	Forward: 5’-GGTACCTGTCACTGGTGGATAAGGA-3’
	Reverse: 5’-CTCGAGGCCTCGAGACCGCTACTCACAGATA-3’
-838/79	Forward: 5’-GGTACCTGTCTACAGAATCCTGATGG-3’
	Reverse: 5’-CTCGAGGCCTCGAGACCGCTACTCACAGATA-3’
-537/79	Forward: 5’-GGTACCCTGGAGGCAATGGACTTG-3’
	Reverse: 5’-CTCGAGGCCTCGAGACCGCTACTCACAGATA-3’
-293/79	Forward: 5’-GGTACCGTCAGTCATGGAGACAGG-3’
	Reverse: 5’-CTCGAGGCCTCGAGACCGCTACTCACAGATA-3’

Table S2. Sequences for primers used in PCR

Gene	Primers
-2441/317	Forward: 5’-GGTACCGGCTACTGGCTTGATTCTTG-3’
	Reverse: 5’-CTCGAGCGTCTTGGCTCTGACTTC-3’
-1091/317	Forward: 5’-GGTACCCATCTGTATGCCTGGATTAAG-3’
	Reverse: 5’-CTCGAGCGTCTTGGCTCTGACTTC-3’
-824/317	Forward: 5’-GGTACCGGAGTGGTCAGGAATCTG-3’
	Reverse: 5’-CTCGAGCGTCTTGGCTCTGACTTC-3’
-539/317	Forward: 5’-GGTACCAACACCACTTTGGGCTTT-3’
	Reverse: 5’-CTCGAGCGTCTTGGCTCTGACTTC-3’
-232/317	Forward: 5’-GGTACCATCATTCAAGAAGGCAGCT-3’
	Reverse: 5’-CTCGAGCGTCTTGGCTCTGACTTC-3’

Table S3. Sequences for primers used in RT-PCR

**Gene**	**Primer**
CYP3A29	Forward: 5’-TTGCTGGCTACGAGACCACTAG -3’
	Reverse: 5’-CTGCTGGACATCAGGGTGAGT-3’
PXR	Forward: 5’-CAGACAACAGTGGGAAAGAGATCTT-3’
	Reverse: 5’-ATGCCTTTGAACATGTAGGTTGAC-3’
RXR-α	Forward: 5’-GAGGGCTGCAAAGGCTTCT-3’
	Reverse: 5’-ACCGTGCAGTCCTTGTTGTC-3’
β-actin	Forward: 5’-TCTGGCACCACACCTTCT-3’
	Reverse: 5’-TGATCTGGGTCATCTTCTCAC-3’

Table S4. Sequences for SiRNA.

**Gene**	**Sequences**
SiPXR	5’-GCCACUGGUUAUCAUUUCATT-3’
	5’-UGAAAUGAUAACCAGUGGCTT-3’
Si RXR-α	5’-GACAACAAGGACUGCCUGATT-3’
	5’-UCAGGCAGUCCUUGUUGUCTT-3’
SiTLR1	5’-CCCACAAAGUUACAUCUAUTT-3’
	5’-AUAGAUGUAACUUUGUGGGTT-3’
SiTLR2	5’-GCCCUUCCUACACACUUUATT-3’
	5’-UAAAGUGUGUAGGAAGGGCTT-3’
SiTLR4	5’-CAGGAAUCCUGGUCUAUAATT-3’
	5’-UUAUAGACCAGGAUUCCUGTT-3’
SiTLR5	5’-GCUAGAUGUUUCUGGCAAUTT-3’
	5’-AUUGCCAGAAACAUCUAGCTT-3’
SiTLR6	5’-GCCCAAACCUGUAGAAUAUTT-3’
	5’-AUAUUCUCAGGUUUGGGCTT-3’

Supplemental Figure S1-S2

Figure S1: **Effect of cecropin B treatment on cell viability in primary hepatocytes and HepLi cells.** Primary hepatocytes and HepLi cells were treated with 0 ng-2000 ng cecropin B for 24 h. Cell viability was monitored by MTT assay. Results are presented as mean ± SD (n = 4).

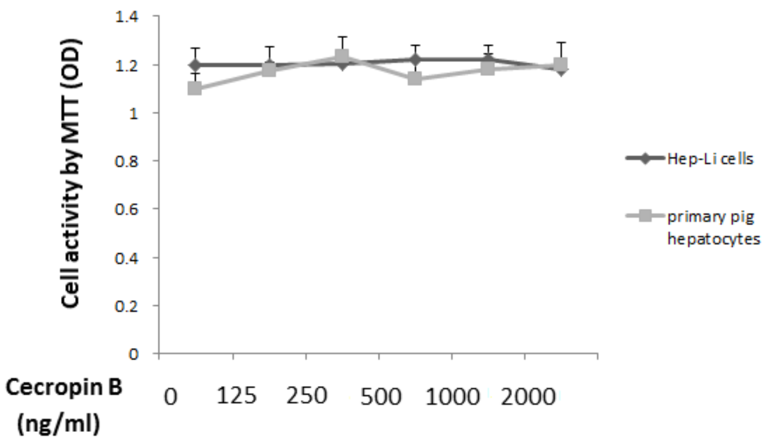

Figure S2. Uncropped, unprocessed images of blots and gels.

Fig.1B

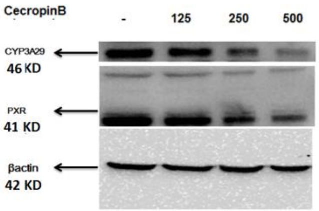

Fig.1D

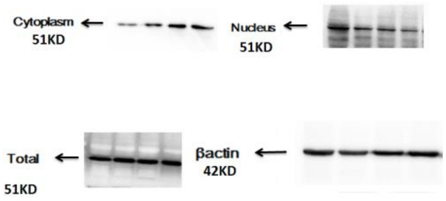

Fig.2A

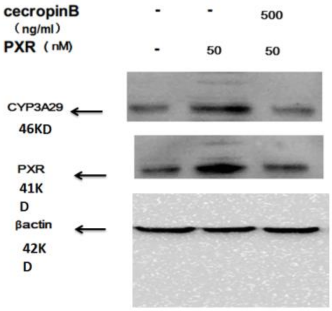

Fig.2B

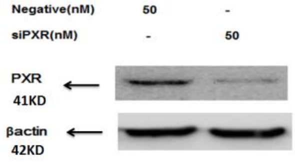

Fig.2C

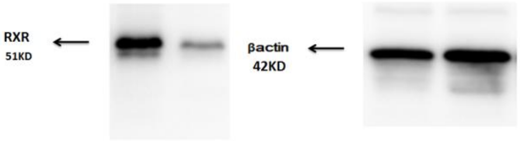

Fig.4B

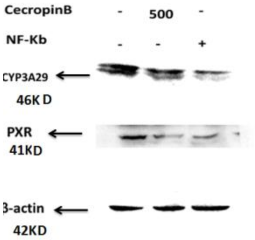

Fig.4C

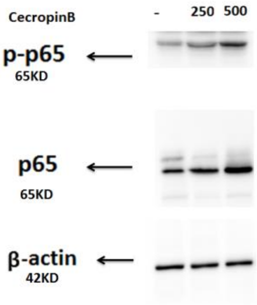

Fig.5C

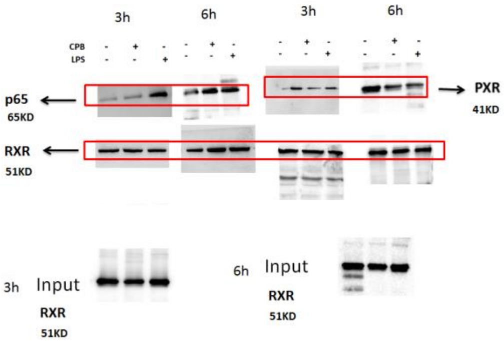

Supplement: Supplementary Information [file srep27876-s1.pdf]
